# Single-Shot Versus Continuous Interscalene Block for Postoperative Pain Control After Shoulder Arthroplasty: A Prospective Randomized Clinical Trial

**DOI:** 10.5435/JAAOSGlobal-D-19-00014

**Published:** 2019-06-11

**Authors:** Samer S. Hasan, Robert H. Rolf, Alexandra N. Sympson, Kathryn Eten, Thomas R. Elsass

**Affiliations:** From the Orthopaedic Surgery (Dr. Hasan), MercyHealth/Cincinnati SportsMedicine and Orthopaedic Center; the Orthopaedic Surgery (Dr. Rolf), Beacon Orthopaedics & Sports Medicine; the TriHealth Hatton Research Institute (Ms. Sympson), TriHealth Good Samaritan Hospital; the Good Samaritan Hospital Orthopedic Center of Excellence (Ms. Eten), TriHealth Good Samaritan Hospital; and the Anesthesiology (Dr. Elsass), Seven Hills Anesthesia, LLC, TriHealth Good Samaritan Hospital, Cincinnati, OH.

## Abstract

**Introduction:**

Continuous catheter infusion of local anesthetics extends the efficacy of regional anesthesia after prosthetic shoulder surgery. Our purpose was to compare continuous interscalene block (CIB) with single-shot interscalene block, and the hypothesis was these would offer similar safety and efficacy in patients with prosthetic shoulder arthroplasty.

**Methods:**

Seventy-six patients were randomized to ropivacaine single-shot interscalene block or CIB after prosthetic shoulder arthroplasty. Postoperative pain scores and opioid use, hospital length of stay (LOS), adverse events, and catheter tip withdrawal were recorded.

**Results:**

Pain scores (*P* = 0.010) and opioid use (*P* = 0.003) on the first postoperative day were lower in the CIB group, but there was no difference in LOS. Adverse events were more common in the CIB group and 10% of catheters pulled out prematurely.

**Conclusion:**

Opioid use and pain levels during first postoperative day are clinically less after CIB, but this did not shorten LOS. The benefits of CIB may not justify the higher cost and complication rate.

Postoperative pain after prosthetic shoulder arthroplasty (PSA) affects patient satisfaction and hospital length of stay (LOS), making optimal pain management a priority focus for healthcare providers and essential for creating healthcare value.^1,2^ Regional anesthesia has long been a cornerstone of multimodal perioperative pain management strategy for PSA and has been shown to reduce baseline pain levels, increase patient satisfaction, and decrease hospital stay.^3-5^ Moreover, the efficacy of regional anesthesia can be extended by continuous catheter infusion of local anesthetics.^3,6,7^ However, safety issues surrounding the use of continuous regional anesthesia remain a concern.^8-10^

Although several randomized controlled trials have compared single-shot interscalene block (SSIB) and continuous interscalene block (CIB) for pain management after shoulder surgery,^6,11,12,13,14,15,16,17,18^ we are unaware of any randomized controlled trial comparing SSIB and CIB exclusively in patients undergoing PSA. Ilfeld et al^19^ reported on a retrospective case-control study evaluating the effect of CIB on immediate postoperative rehabilitation after PSA, but safety and analgesic efficacy were not the focus of their study. The purpose of this study was to compare CIB with SSIB for postoperative pain control after shoulder arthroplasty. The hypothesis being that either of the two analgesic interventions would offer similar safety and efficacy.

## Methods

This study was an institutional review board-approved, prospective, randomized, controlled clinical trial registered with the NIH (NCT02267044) and conducted at a single community hospital in Cincinnati, Ohio, that performs over 150 prosthetic shoulder arthroplasties annually. All patients 18 years or older undergoing PSA, reverse total shoulder arthroplasty, or hemiarthroplasty by one of two fellowship-trained shoulder surgeons were considered for this randomized controlled trial. Patients were excluded if they had a body mass index of greater than 40 kg/m^2^, an American Society of Anesthesiologist class 4 physical status or greater, a history of drug or alcohol abuse, an allergy to ropivacaine, any coagulation disorders, existing nerve injury, severe bronchopulmonary disease, or if they were oxygen dependent.

**Figure 1:**
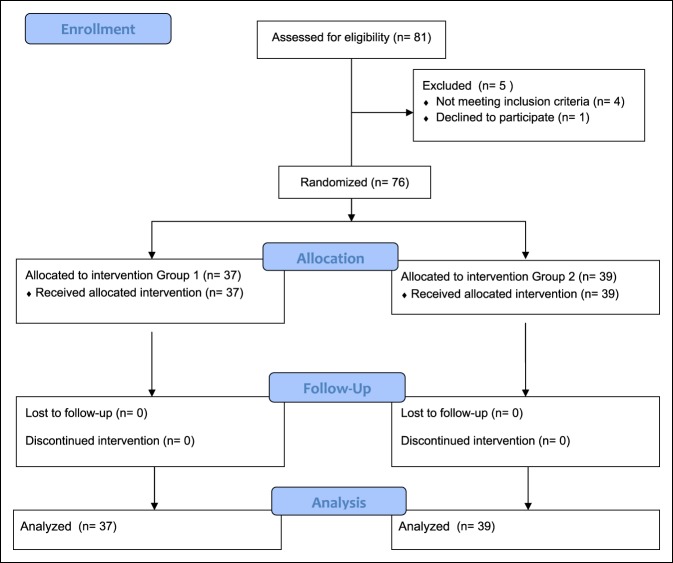
Flowchart demonstrating enrollment and randomization scheme.

Eighty-one patients, from July 2014 to October 2015, consented to participate in this study (Figure 1), but five of these patients were withdrawn from the study for various reasons, including inability to be randomized to CIB because of current health status and comorbidities (n = 4) and withdrawal of consent by the patient on the day of surgery (n = 1). The remaining 76 patients were randomized by the study coordinator in a 1:1 ratio by permuted mixed block size randomization table to either group 1 (n = 37) receiving SSIB (control group or group 2 (n = 39) receiving CIB. Patients presented with primary glenohumeral osteoarthritis (n = 5), secondary glenohumeral osteoarthritis (n = 58), comminuted 3- or 4-part closed fracture of the proximal humerus (n = 2), rotator cuff arthropathy (n = 8), or a failed shoulder arthroplasty (n = 3). All patients completed follow-up, and data collection was completed by October 2015.

All patients underwent preoperative ultrasonography-guided regional anesthesia. Patients undergoing SSIB received a one-time dose of 30 mL, 0.5% preservative-free ropivacaine. Patients undergoing CIB received a single injection of 30 mL, 0.5% preservative-free ropivacaine followed by threading an 18-gauge open-tip stimulating catheter under ultrasonography guidance.^12,13^ After confirmation of catheter tip placement, patients received 0.2% preservative-free ropivacaine at 8 mL/hr beginning at the conclusion of surgery and delivered for approximately 50 hours (or finish of 400 mL) by means of a catheter attached to an elastomeric infusion system (OnQ Pain Relief System: Select A Flow, Kimberly–Clark). The catheter was secured to the skin using Dermabond (Ethicon US, LLC, Johnson & Johnson) and Tegaderm (3M) in all cases. Patients were instructed how to remove the empty pain ball when empty, approximately 50 hours after surgery.

Patients underwent preoperative assessments and daily postoperative pain and satisfaction queries for the first 4 days after surgery as well as on the 10th day (±5 days) after surgery. Primary outcomes included 10-point VAS score to assess pain, a 5-point Likert scale to score overall satisfaction, and postoperative opioid consumption measured in morphine sulfate equivalent to facilitate comparisons. In addition, secondary outcomes recorded were the hospital LOS (in days), instances of unanticipated catheter tip withdrawal, and adverse events arising perioperatively. Finally, the mean cost of both SSIB and CIB was determined.

Demographic variables were compared using the independent samples *t*-test, Pearson chi square test, or Fisher exact test, as appropriate. The Mann-Whitney *U* test was used for non-normally distributed variables, and the univariate chi square analysis or Fisher exact test, as appropriate, was used for categorical variables. Group effect size was calculated using group 2 as the control group and the pooled SD. At 80% power and alpha level of 0.05, a priori power analysis was conducted with Statistical Solutions nQuery Advisor 2.0. Previous studies,^6^ specifically by Klein et al,^17^ that examined CIB versus SSIB for biceps tenodesis and open rotator cuff repair were used to determine that seven patients would be needed in each group. However, to be conservative, the study by Borgeat et al^11^ was used to calculate the sample size, which determined that closer to 48 patients per group would be required at alpha 0.05 and 80% power, although this study was less comparable to our study. Based on these studies, the final study sample size of n = 76 patients, with 38 per group was determined to be appropriate. The power analysis did not incorporate a drop-out rate because the interventions are considered standard practice, and the follow-up period for this study was brief. Statistical analysis was carried out using IBM SPSS Statistics for Windows version (IBM). A CONSORT checklist was completed after the completion of the study.

## Results

Thirty-five of the 76 patients were women and 41 were men, and the mean age was 68.6 years (SD 8.8 years). Fifty patients (65.8%) underwent anatomic total shoulder arthroplasty, 21 patients underwent reverse shoulder arthroplasty, and 5 patients underwent either resurfacing or stemmed hemiarthroplasty. No differences between the groups were detected at alpha = 0.05 level and 95% confidence interval for any of the demographic variables, including age, sex, body mass index, comorbidities, American Society of Anesthesiologist level, diagnosis, and duration of surgery (Tables 1 and 2). Four of 39 patients (10.3%) receiving catheters for continuous infusion subsequently had their catheters pulled out prematurely; however, these patients remained in the CIB group because of intent to treat.

**Table 1:**
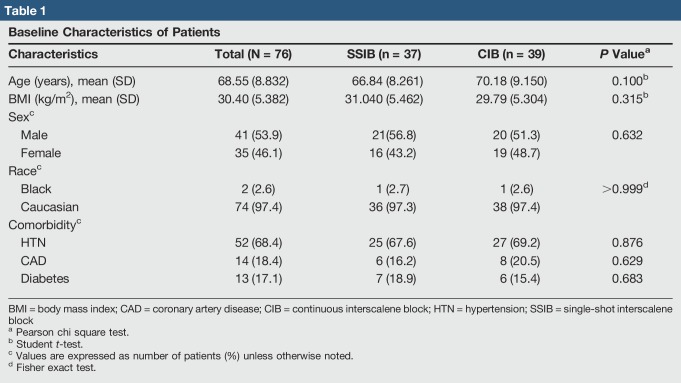
Baseline Characteristics of Patients

**Table 2:**
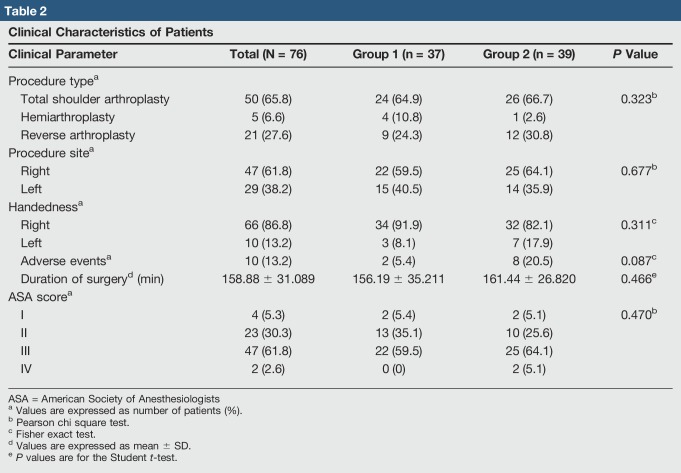
Clinical Characteristics of Patients

**Figure 2:**
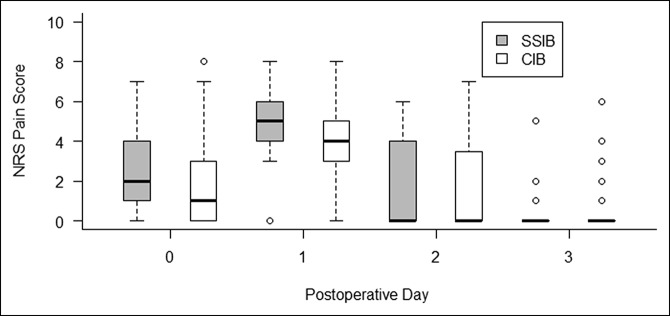
Chart showing NRS pain scores in the SSIB and CIB group for postoperative days 0 through 3. CIB = continuous interscalene block; NRS = numeric rating scale; SSIB = single-shot interscalene block.

**Figure 3:**
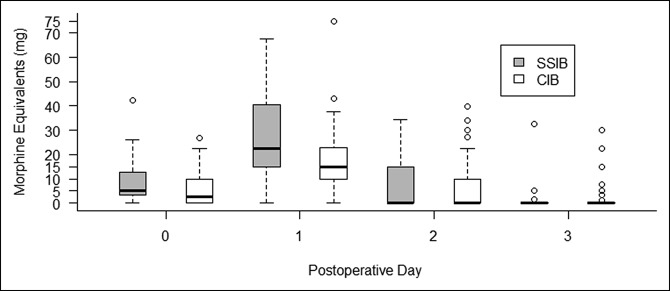
Chart showing opioid consumption in morphine equivalents (mg) for postoperative days 0 through 3. CIB = continuous interscalene block; SSIB = single-shot interscalene block.

Pain scores and opioid use on the first postoperative day were significantly lower in the CIB group (*P* = 0.010, *P* = 0.003, respectively), but these subsequently normalized (Figures 2 and 3). Postoperative pain scores and opioid use are shown in Tables 3 and 4 with effect size reported in Table 5. There was no difference in hospital LOS between the SSIB group (median = 1.00, IQR 1.00) and the CIB group (median = 2.00, IQR 1.00, *P* = 0.404) (Table 6). Opioid use and pain scores were highly correlated on the day of surgery (PACU) with a correlation coefficient of 0.785 and *P* value < 0.001 as well as on the first postoperative day (0.370, *P* value = 0.001) and POD #2 (0.867, *P* value < 0.001). A single patient in the SSIB group who had been on long-term preoperative opioids was identified as an outlier; repeating the analysis without this patient's data did not markedly change the results and so the data were retained.

**Table 3:**
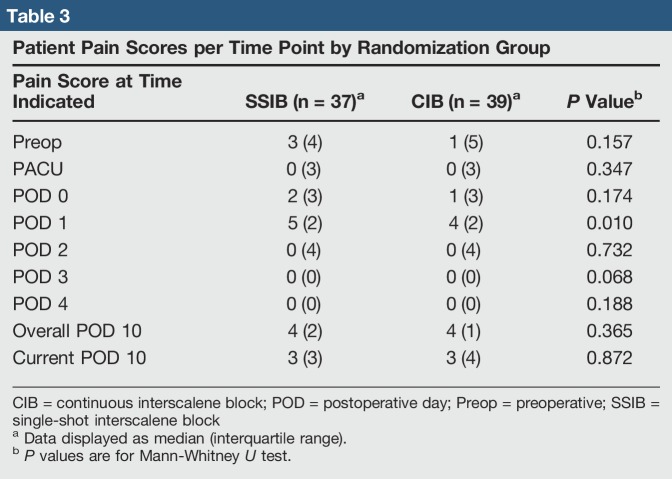
Patient Pain Scores per Time Point by Randomization Group

**Table 4:**
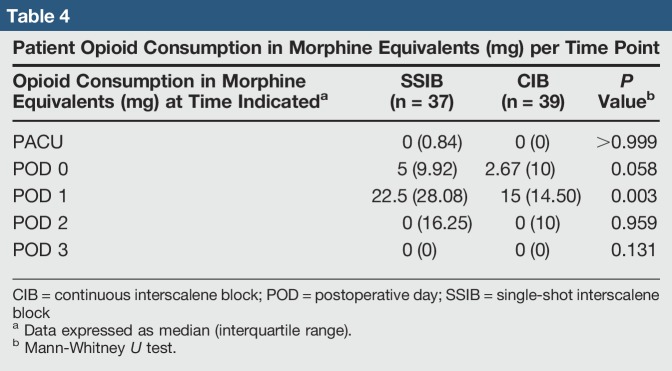
Patient Opioid Consumption in Morphine Equivalents (mg) per Time Point

**Table 5:**
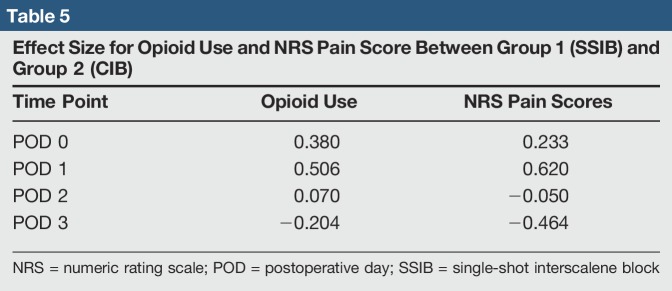
Effect Size for Opioid Use and NRS Pain Score Between Group 1 (SSIB) and Group 2 (CIB)

**Table 6:**
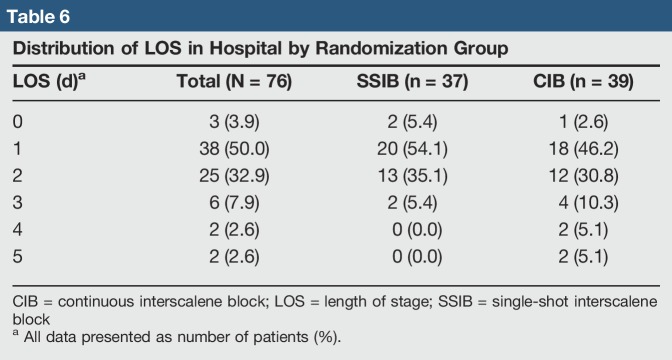
Distribution of LOS in Hospital by Randomization Group

Several adverse events related to regional anesthesia were noted in both groups as shown in Table 7. Adverse event rates were higher in the CIB group (8/39 versus 2/37) but this difference was not statistically significant with the numbers available (*P* = 0.087). The most common adverse event in the CIB group was three syncopal episodes, one of which resulted in pacemaker implantation. However, there were no instances of pneumothorax or catheter tip breakage.

**Table 7:**
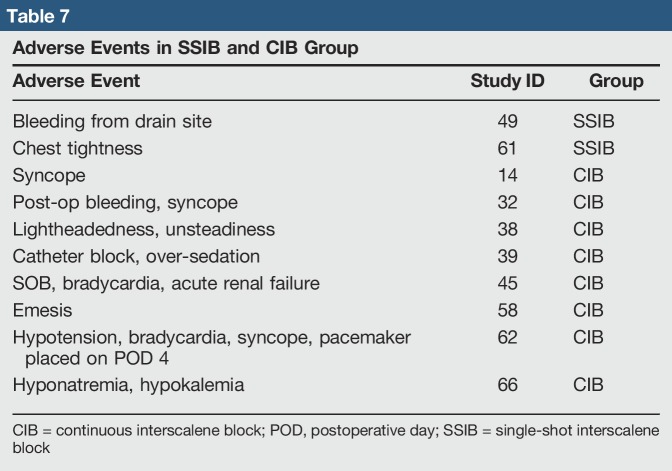
Adverse Events in SSIB and CIB Group

The itemized costs of both SSIB and CIB are noted in Table 8. The cost of the elastomeric infusion system and the additional local anesthetic resulted in a higher cost for CIB by more than $450 per case.

**Table 8:**
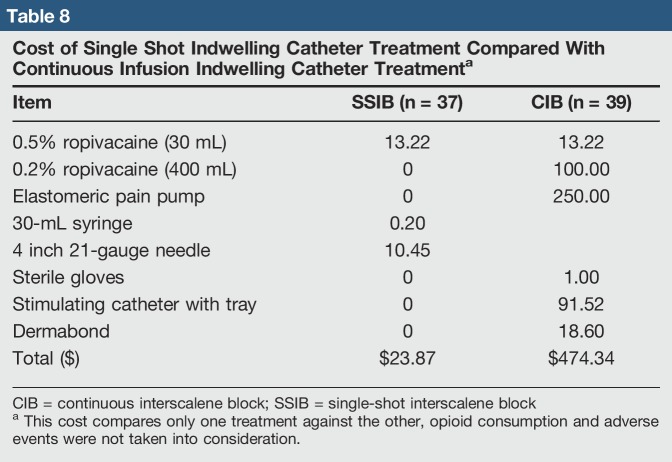
Cost of Single Shot Indwelling Catheter Treatment Compared With Continuous Infusion Indwelling Catheter Treatment^a^

## Discussion

Our study demonstrates that pain levels and opioid use during the first day postoperatively after CIB were statistically less than after SSIB (*P* = 0.010 and *P* = 0.003, respectively; Tables 3 and 4). Although this is a clinically notable outcome,^20^ it did not translate to a shorter hospital LOS (Table 6). Many studies comparing SSIB and CIB have not specifically evaluated LOS.

In contrast, more recent randomized controlled studies comparing CIB with other postoperative pain management strategies have explored the relationship between postoperative pain levels and LOS. One study demonstrated improved pain levels with CIB compared with liposomal bupivacaine, but without impacting LOS.^21^ Another study comparing similar treatment groups found that patients receiving CIB had increased pain levels and a longer LOS,^22^ whereas a third study found that both treatment groups had similar pain levels and similar LOS.^4^ In our study, LOS may have been prolonged due to extraneous factors such as inadequate family support at home requiring transfer to a skilled nursing facility or inefficient transfer to skilled nursing facility on weekends, and as such cannot be critically evaluated.

Interscalene catheter placement has been recognized as technically challenging, which may explain the relatively slow growth in the use of this technique.^23,24^ This study demonstrates that experienced anesthesiologists at a community hospital are able to insert interscalene catheters reproducibly and without serious complications. However, the potential for serious complication remains^6^ and even centers with great experience in regional anesthesia have reported serious complications including pneumothorax and intravascular injection,^25,26^ as well as transient postoperative neurological symptoms. The latter are relatively common the first few days postoperatively, but infrequently these persist after the first month, and very rarely past 6 months.^25,27^ Our study found that the overall rate of adverse events is greater after CIB than after SSIB. One patient required pacemaker implantation after syncopal episodes that may have been related to inadvertent intravascular injection of local anesthetics during CIB. However, none of the patients in this study developed a pneumothorax or brachial plexus injury (Table 7).

Comparing the cost of treatment was not an original objective of this study. However, we estimated the itemized costs incurred for both SSIB and CIB (Table 8) and found the CIB cost to be approximately $450 more than SSIB because of the cost of the catheter tray, elastomeric pump, and additional ropivacaine. In addition to higher baseline costs, the increased number of adverse events likely added to the overall cost of CIB, although we were unable to quantify this in our study. Further study is needed to determine whether the lower pain scores and opioid consumption on POD 1 offset the additional financial burden and clinical risk of CIB.

Catheter fixation is essential for optimal effectiveness of CIB,^6^ but this can be difficult about the shoulder because of its mobility and the proximity of the surgical field to the catheter entry site. Our study found a 4 of 39 (10.3%) rate of catheter tip withdrawal, despite securing the catheter entry site and sealing it with an occlusive dressing and efforts to avoid placing surgical drapes over the entry site. A recent study reported a 5 of 33 (15.2%) rate of catheter tip withdrawal after CIB,^22^ comparable to the incidence reported here, highlighting the challenge of securing the catheter after PSA. Catheter tip withdrawal influences the duration of action and overall efficacy of CIB; its rate remains too great and further efforts to reduce this are needed.

Several recent studies have compared interscalene nerve block with emerging perioperative pain management interventions such as liposomal bupivacaine and various cocktail infiltrations. Okoroha et al^4^ found an increase in early postoperative pain with liposomal bupivacaine and an increase in opiate analgesic use at the end of the day of surgery after SSIB from a prospective randomized trial. In a retrospective cohort study comparing SSIB with and without preoperative intravenous dexamethasone and intraoperative infiltration of liposomal bupivacaine, Routman et al^28^ found that the addition of liposomal bupivacaine and dexamethasone reduced postoperative pain and hospital LOS after shoulder arthroplasty, although the authors were unable to differentiate between the effects of the liposomal bupivacaine and dexamethasone. In a randomized prospective study, Sabesan et al^22^ compared liposomal bupivacaine and CIB for shoulder arthroplasty. The authors found no difference in LOS and an increased number of complications and cost for CIB and concluded that liposomal bupivacaine appears to be equivalent to CIB in terms of pain relief, narcotic usage, length of hospital stay, and time until first narcotic rescue.^22^ Abildgaard et al^21^ found that patients receiving CIB had decreased opioid consumptions and pain scores than those receiving liposomal bupivacaine. However, patients receiving liposomal bupivacaine were not bridged with SSIB until the liposomal bupivacaine took effect,^21^ which is in contrast to patients in the study by Sabesan et al.^22^ Further studies will be needed to reinforce these findings and to compare CIB with alternative perioperative pain management interventions and may offer an additional avenue of investigation providing effective pain control at comparable safety and cost.

A study limitation includes the fact that cost was not an original focus of the study, and that the cost of adverse events was not calculated. Another limitation is the nonblinded nature of the study, which was due to the threaded catheter in the CIB group. The use of the VAS, a one-dimensional analog rating scale, is a potential limitation because it has limited ability to detect subtle changes and because patients tend to report high scores.^29,30^ As mentioned, the single patient on long-term preoperative opioid treatment should have been excluded from this study although this did not markedly alter the study results. Patients undergoing revision surgery and fracture surgery may have different postoperative pain levels than patients who underwent primary total shoulder arthroplasty and as such should probably have been excluded from this clinical trial. In retrospective analysis, by excluding the three revision cases in this study population, the opioid consumption on POD #0 was statistically significant between groups (U = 467.500, *P* = 0.026), with the SSIB group having greater morphine consumption (median = 5.00, IQR = 9) than the CIB group (median = 2.36, IQR = 10). The findings of the retrospective analysis were otherwise unchanged. Strengths of this study include the use of a consistent nerve block technique and the randomized controlled study design.

## Conclusion

Continuous interscalene nerve block substantially reduced opioid use (*P* = 0.003) and pain scores (*P* = 0.010) during the first day postoperatively compared with single-shot interscalene nerve block. However, the complication rate was higher after CIB and LOS in the hospital was longer, although this was determined to not be statistically significant in this study (*P* = 0.404). Further study will be necessary to determine whether the benefits of CIB justify the higher cost and overall complication rate.
